# Cross-talk between MYOC p. Y437H mutation and TGF-β2 in the pathology of glaucoma

**DOI:** 10.7150/ijms.43614

**Published:** 2020-04-27

**Authors:** Yang Yang, AL Sabri Waled Abdulghani Abdulatef, LuSi Zhang, Haibo Jiang, Zhou Zeng, Haibo Li, Xiaobo Xia

**Affiliations:** 1 Eye center of Xiangya Hospital, Central South University, Changsha 410008, Hunan Province, China; 2 Department of Ophthalmology, the First People's Hospital of Yueyang, Yueyang, 414000, Hunan Province, China; 3 The School of Life Sciences, Central South University, Changsha 410078, Hunan Province, China; 4 Hunan Key Laboratory of Ophthalmology, Central South University, Changsha 410008, Hunan Province, China

**Keywords:** Glaucoma, *MYOC*, Aqueous humor, TGF-β2

## Abstract

**Objective**: To identify the interaction between the MYOC Y437H mutation and TGF-β2 in a family with primary open-angle glaucoma (POAG).

**Methods**: The MYOC Y437H mutation was identified in a family with POAG; the family was a fourth-generation family with 27 members, of which 6 members were affected. Analysis focused on the secreted myocilin protein and TGF-β2 found in the aqueous humor. Samples were taken both from normal controls and MYOC mutant carriers and cross-talk between MYOC Y437H and TGF-β2 were evaluated in the trabecular meshwork (TM) cells.

**Results**: Aqueous humor secreted myocilin protein levels were reduced while TGF-β2 levels were increased in patients with the *MYOC* (c.1309T>C) mutation. This inverse relationship indicated that elevated TGF-β2 may be an important pathogenic mechanism in the progression of myocilin-related POAG. In TM cells expressing the MYOC Y437H mutant, exogenous TGF-β2 also significantly increased myocilin expression as well as the endoplasmic reticulum (ER) stress markers GRP94 and CHOP. This increase in TGF-β2 was also associated with increased cell death in cells carrying the MYOC Y437H mutation.

**Conclusion**: These data collectively suggest that the mutual interaction between glaucomatous *MYOC* mutation and TGF-β2 contributed to the cell death of TM cells. This relationship also provides a new, therapeutic targets for the treatment of glaucoma.

## Introduction

Glaucoma is a kind of optic nerve disease which causes symptoms such as excavation of optic disc and visual field defect. Increased intraocular pressure is a risk factor for developing glaucoma. Critically, it is the second leading cause of blindness in developed countries 1. Primary open-angle glaucoma (POAG) is the most common type of glaucoma, accounting for 60%-70% of glaucoma cases. In its typical presentation, POAG usually affects both eyes—but not necessarily in a symmetrical way. Regarding its onset, POAG occurs across a wide age range, but is most commonly diagnosed at the age of 40^2^. Important risk factors for POAG include genetics, age, and environment. Previous reports have shown that gene mutations in *MYOC*, *OPTN*, and *WDR36* are associated with the incidence of POAG 3-5. Recently, we studied a large family from Hunan in China that had several members suffering from POAG. The family was fourth-generation with 27 members, of which 6 members had POAG. We used PCR and direct Sanger sequencing to determine any underlying genetic factors and found that an autosomal dominant gene *MYOC* (c.1309T>C) mutation was the pathogenic gene for the development of POAG in this family. Furthermore, up-regulation of TGF-β2 protein levels was detected in the aqueous humor of patients with this specific *MYOC* (c.1309T>C) mutation. Finally, we found that exogenous TGF-β2 treatment enhanced MYOC Y437H mutation-induced endoplasmic reticulum (ER) stress and resulted in increased cell death. Collectively, these results suggest a molecular mechanism for the cross-talk between MYOC Y437H and TGF-β2 in the pathology of glaucoma.

## Materials and methods

### Study subjects

 A family manifesting the signs and symptoms of POAG was diagnosed at the Xiangya 1^st^ Affiliated Hospital in Changsha, Hunan. The family included 15 males and 12 females, of which four male and two female members were affected with PAOG. All the subjects included in the study signed informed consent approved by Changsha Xiangya Hospital Ethics Committee, and were diagnosed according to the POAG diagnostic criteria drafted in 1987 by Glaucoma Group, Commission of Ophthalmology, Chinese Medical Association.

### Clinical examination

The proband and other members of the family took a physical and ophthalmologic examination, which included visual acuity testing, intraocular pressure (IOP), visual field, slit lamp examination and ultrasound biomicroscopy (UBM).

### Mutation detection

DNA extraction was performed following the standard phenol/chloroform extraction method as previously described 6. After dissolving, the concentration and purity of gDNA samples were measured using a NanoDrop ND-1000 spectrophotometer (Thermo Scientific, Madison, WI) and underwent appropriate standardization process.

According to the *MYOC* sequence, Primer 3 online software was used to design the primers. The designed primers were listed in **Table 1**, and primers were synthesized by Shanghai Sangon Biotech Co., Ltd (Shanghai, China). The PCR system (10 μl) was made up of 50 ng DNA template, 30 ng upstream/ downstream primer, 5 μl doubled Premix Ex Tap TM enzyme (Takara, Japan), and 2 μl PCR water. PCR reaction was carried out in a 96-well plate heat circulator (Apllied Biosystem, USA). The reaction conditions used were : i) 95˚C pre-degeneration for 5 min; ii) 95˚C degeneration for 30 sec; annealing for 58-63˚C for 30sec (4 primers were completed in the same reaction); extension at 72˚C for 30 sec; total 32circles; iii) extension at 72˚C for 7 min, and the samples were stored at 4˚C.

The PCR products of the DNA samples collected from the family underwent Sanger sequencing by Boshang Biotech Co., Ltd (Shanghai, China). The sequencing results were analyzed using SeqMan program of DNA STAR software (DNASTAR Inc., Madison, WI) and compared with the reference sequence in the UCSC database to screen for mutations.

### ELISA, Immunoblotting and Immunostaining

Aqueous humor was collected and the levels of human TGF-β2 were measured using ELISA kit (ab100648, Abcam, Cambridge, MA, UK) according to instructions provided. Immunoblotting and immunostaining were performed as described previously 7, 8. The primary antibody used to detect myocilin was obtained from Santa Cruz (sc-137233, Santa Cruz Biotechnology, Santa Cruz, CA), while the transferrin antibody was from Abcam (ab82411). Recombinant human TGF-β2 protein was obtained from Thermo Scientific (PHG9124, Thermo Scientific, Madison, WI), dexamethasone was obtained from Sigma (D4902, Sigma, St Louis, MO). The signal intensities of both the myocilin and transferrin bands were analyzed by Image J (National Institutes of Health, Bethesda, MD). The ratio of myocilin relative to the loading control transferrin was first calculated, after which the relative expression was expressed as a percentage of the mean using the control sample as reference.

### TM Cell Culture

Human TM cells were isolated from dissected human TM tissue explants derived from nonglaucomatous donors (NTM). All donor tissues were obtained and managed according to the guidelines in the Declaration of Helsinki for research involving human tissue and also approved by Ethics Committee of Xiangya Hospital, Central South University. Human TM cells were isolated and grown as described previously 9, 10. Briefly, the TM tissue was dissected and dissociated with 0.05% Trypsin for 20 min. After centrifugation, the TM cells were grown in DMEM containing 2 mM L-glutamine, penicillin (100 U/ml), streptomycin (0.1 mg/ml), and 10% FBS at 37ºC in a 5% CO_2_ humidified incubator. All of these reagents were obtained from Thermo Scientific.

To construct the vector overexpressing *MYOC*, a *MYOC* cDNA (OriGene Technologies, Rockville, MD, USA) was amplified and cloned into lentiviral vector LV6 vector plasmid. The *MYOC* Y437H mutant vector was generated using a QuikChange® Site-Directed Mutagenesis Kit (Agilent, Santa Clara, CA, USA), and the mutant primer was designed using the QuikChange® Primer Design Program (https://www.genomics.agilent.com/primerDesignProgram.jsp). Lentiviral supernatant was produced by co-transfection of LV6-WT MYOC or Y437H mutant vector into 293T cells with lentiviral packaging plasmid. For vector transfection, TM cells (passage 3) were cultured in 60 mm dishes to a density of approximately 7×10^5^ cells. Cells were then infected for 6 h with lentiviruses carrying either WT or mutant MYOC, after which the media was changed and culturing occurred for another 5 d. After, all cultures were treated with 50 ng/ml of TGF-β2 for 48 h. All experiments were conducted in triplicate.

### Statistical analysis

All statistical analyses were performed using Prism 5 software (GraphPad, San Diego, CA). Two-tailed Student's t test was used to determine the significance of differences between two groups. All data are presented as mean ± SEM. Results were considered significant at* p*<0.05.

## Results

### Clinical evaluation

**Figure 1:**
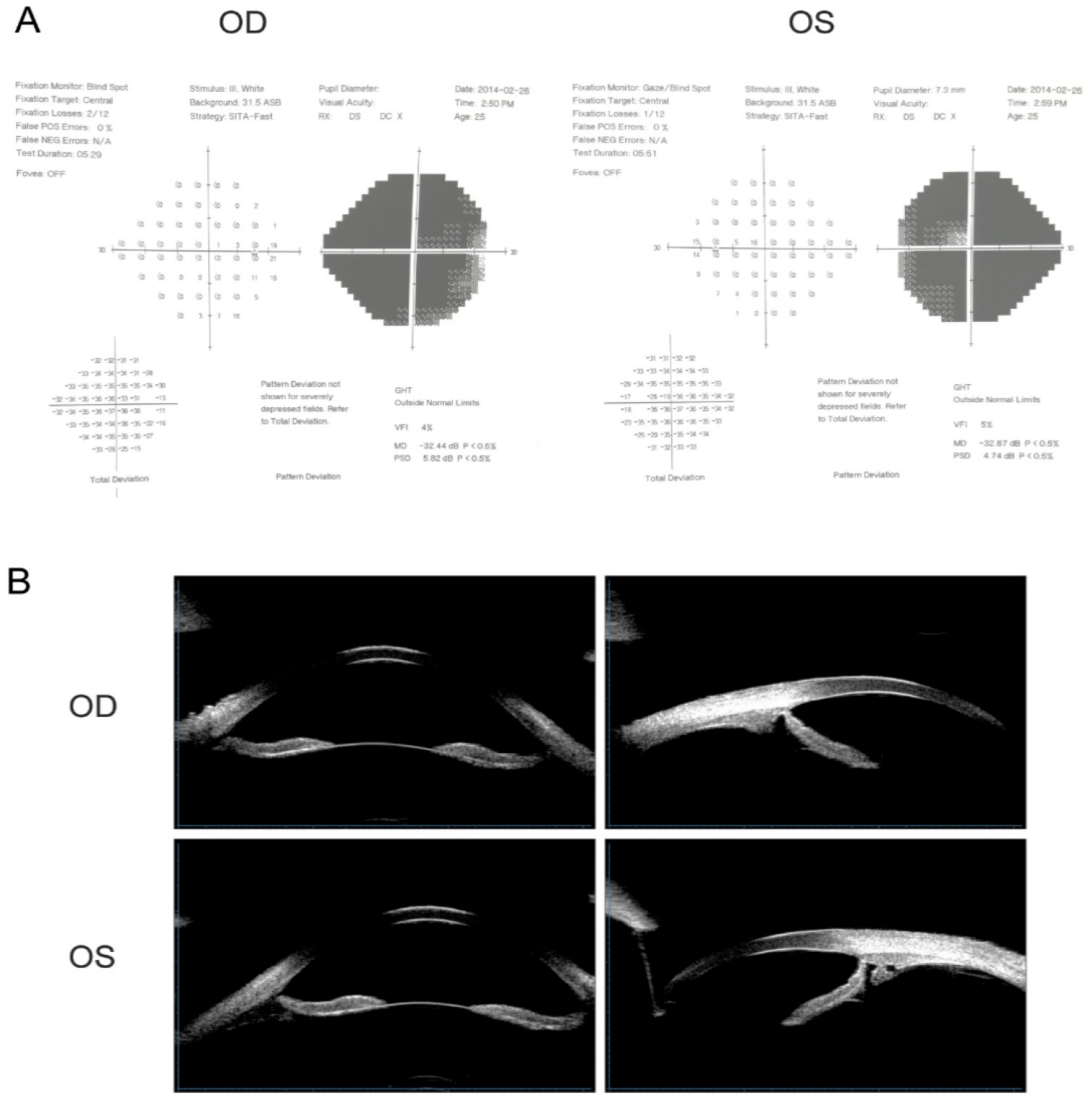
** Visual field examination and UBM detection results of the proband.** (A) Visual field examination of MD were -32.44 dB in the right eye and -32.87 dB in the left eye. Note tubular visual field of left eye. (B) The central depth of the anterior chamber was 3.27mm in the right eye and 3.25mm in the left eye. The iris of both eyes was slightly depressed, and the attachment of the iris root was located at the front without sheltering from the scleral process. The chamber angle was open and ophthalmic lens were in situ.

The proband of the family (Ⅲ:9) was diagnosed with POAG at 16 years of age. After suffering from deteriorating vision in both eyes along with swelling and pain for six months, she visited the hospital to have an ophthalmic evaluation. Visual acuities were 20/500 (0.04) in the right eye (OD) and 20/200 (0.1) in the left eye (OS). IOPs were 25 mmHg in both eyes; cup-to-disc ratio (C/D): OD 0.8, OS 0.9; visual field examination of MD: OD -32.44 dB, OS -32.87 dB. The proband was consistent with a POAG diagnosis, given her high IOP, open irido-corneal angle, optic atrophy, and visual field defect (Fig. 1A). UBM detection results are shown in Fig.1B. After admission and IOP stabilization, a left eye combined trabeculectomy was performed under local anesthesia. Post-operative IOP was controlled at approximately 8 mmHg. The filtering blebs were functional and the anterior chamber depth was normal. The remaining family members were subjected to ocular examinations, and the clinical characteristics of mutation carriers are listed in **Table 2**.

### MYOC gene mutation screening results

**Figure 2:**
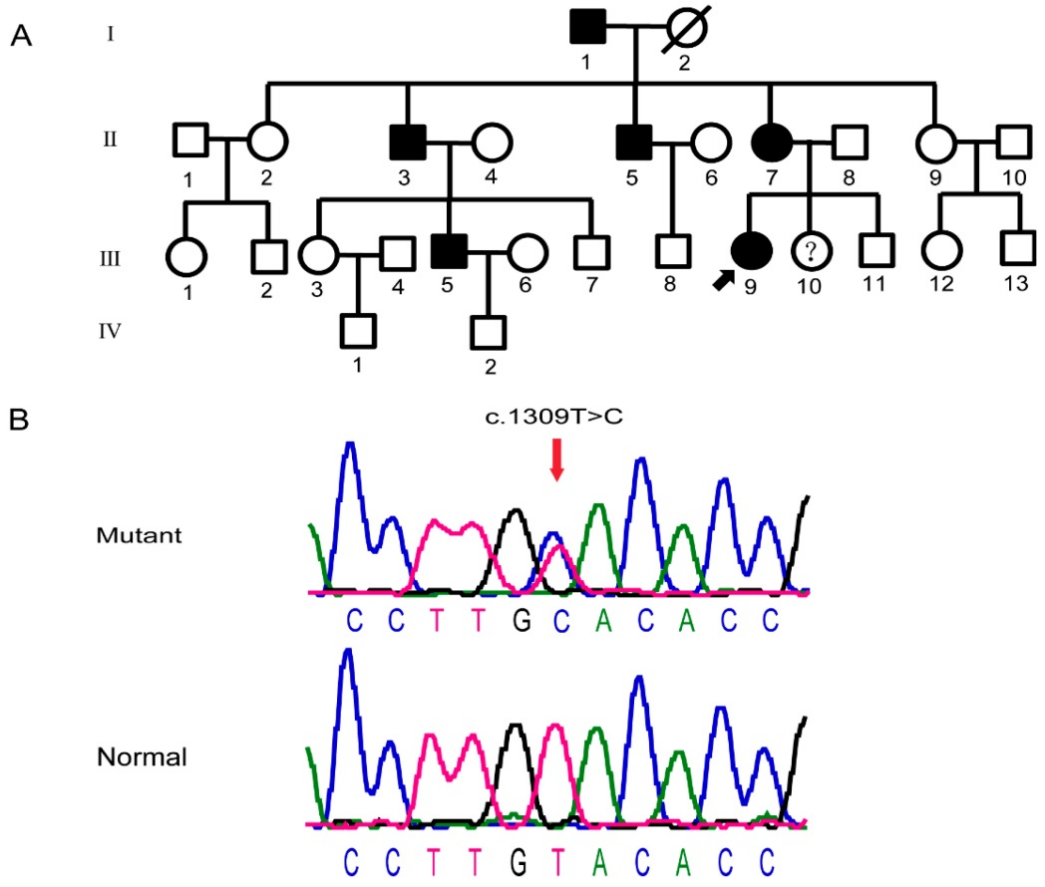
** Pedigree structure and sequence diagram of the POAG family.** (A) The family with POAG pedigree. Roman numerals referred to generations, and individuals within a generation were numbered from left to right. Proband was noted with arrow. Please note III:10 carried Tyr437His mutation, but was not diagnosed with glaucoma. (B) DNA sequence of *MYOC* c.1309T>C mutation. Arrows referred to mutant bases. The upper is the sequence map, and the below is the sequence map of the patients, and the red arrow shows the mutation site.

This particular Chinese pedigree with POAG consisted of 27 members across four generations (Fig.2A). There were no gender difference among the glaucoma patients in the family, which were consistent with its autosomal dominant pattern of inheritance. After detection of the *MYOC* gene, heterozygosity variation of MYOC: c.1309T>C (P.Tyr437His) was found in members Ⅱ:3, Ⅱ:5, Ⅱ:7, Ⅲ:5, Ⅲ:9, and Ⅲ:10. This mutation was not identified in members Ⅱ:2, Ⅱ:9, Ⅲ:3, Ⅲ:7, or Ⅲ:8 (Fig.2B). Given these findings, we considered that the mutation and the disease-related phenotype were co-separated. This mutation was not identified in 100 unrelated normal controls, which excluded the interference of genetic polymorphisms.

### Aqueous humor TGF-β2 levels were elevated in patients with MYOC Y437H mutation

**Figure 3:**
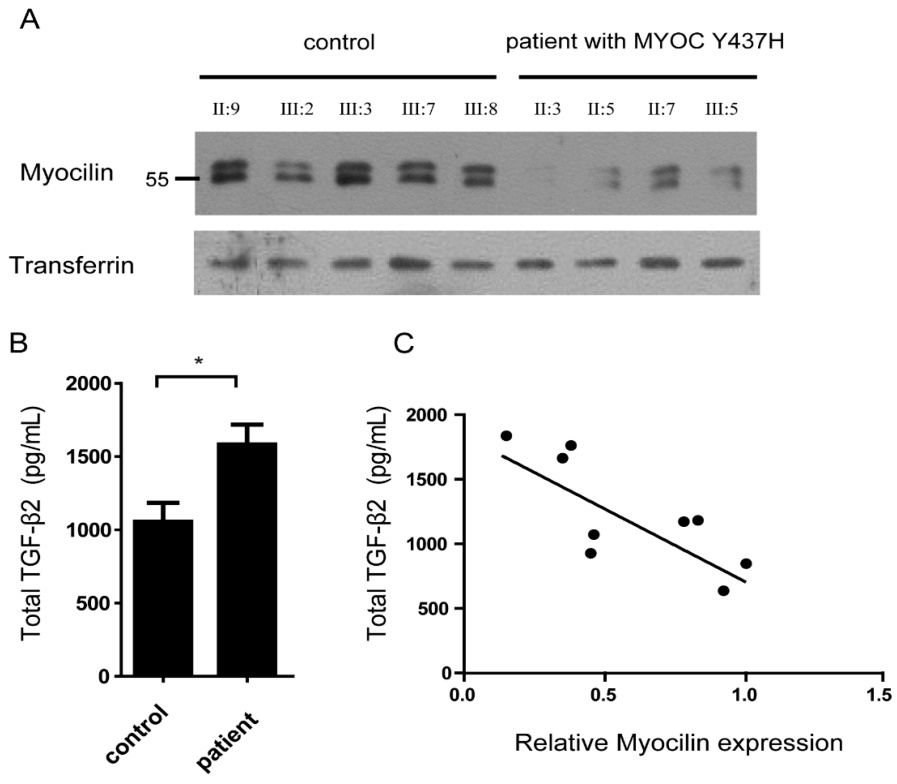
** Aqueous humor TGF-β2 level are elevated in patients with *MYOC* Y437H mutation.** (A) Immunoblotting to detect myocilin and transferrin expression in aqueous humor of normal and *MYOC* Y437H mutation carriers. (B) The secreted TGF-β2 level in the aqueous humor were detected by human ELISA kit. (C) Pearson correlation analysis of myocilin and TGF-β2 expression in aqueous humor. The signal intensities of myocilin and transferrin bands were analyzed by Image J, the relative expression is shown as a percentage of the mean using the control sample as reference.

Myocilin is a secreted protein which can be detected in the aqueous humor. Given this, we next analyzed myocilin protein levels in the aqueous humor of five normal (Ⅱ:9, Ⅲ:2, Ⅲ:3, Ⅲ:7, Ⅲ:8) and four MYOC Y437H mutation carriers (Ⅱ:3, Ⅱ:5, Ⅱ:7, Ⅲ:5) using immunoblotting. In all cases, transferrin was used as the loading control. When compared with normal controls, there was little or no detectable myocilin in the aqueous humor of *MYOC* Y437H mutation carriers, indicating that myocilin secretion was inhibited by the presence of the *MYOC* Y437H mutation (Fig.3A). Previous studies showed that cytokines including TGF-β2 in aqueous humor have critical roles in patients with open-angle glaucoma 11. To test whether the *MYOC* Y437H mutation was involved in cytokine synthesis, secreted TGF-β2 obtained from both the aqueous humor from normal controls and *MYOC* Y437H mutation carrier patients were tested using a human ELISA kit. When compared with the control group, the *MYOC* Y437H mutants had increased TGF-β2 secretion. Specifically, the secreted TGF-β2 of the control and *MYOC* Y437H mutation groups were 1053.6±130.7 ng/ml and 1582.3 ±174.3 ng/ml, respectively (Fig. 3B). We also found a significant, inverse correlation between myocilin and TGF-β2 levels in aqueous humor (R^2^=0.62, P<0.05, Fig. 3C). The difference in TGF-β2 levels in patients with POAG *MYOC* Y437H mutation might relate to the dysregulated secretion of myocilin in aqueous humor. Taken together, these results provide direct evidence demonstrating that the *MYOC* Y437H mutation increased the expression of TGF-β2 in aqueous humor, which might be an important pathogenic mechanism in POAG development.

### TGF-β2 enhances MYOC Y437H mutation induced endoplasmic reticulum(ER) stress

**Figure 4:**
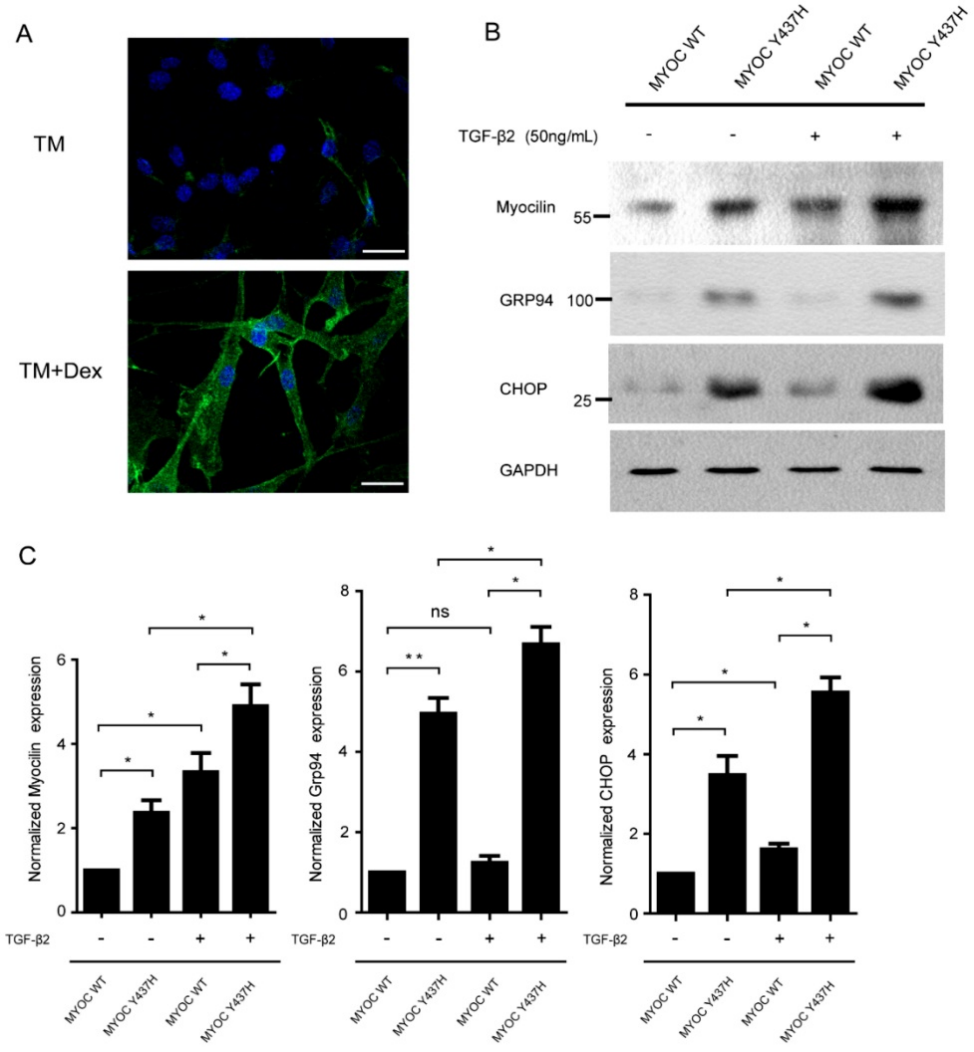
** TGF-β2 enhances MYOC Y437H mutation induced endoplasmic reticulum(ER) stress.** (A) TM cells were exposed to 100nM Dex for 5 days, then subjected to immunostaining of myocilin. Scale bars 30 μm. (B) TM cells were overexpressed with WT MYOC or Y437H mutant first, then exposed to TGF-β2 (50ng/ml) for 48h. Protein from cell lysates were subjected to SDS-PAGE and immunoblotted with the antibodies indicated. (C) Quantification of myocilin, Grp94 and CHOP levels. Relative protein levels were calculated using image J software. The results were obtained from three independent experiments.

As the expression of myocilin can be induced by dexamethasone (Dex) in TM cells, we next characterized isolated primary human TM cells by their response to Dex 12. As given in Fig. 4A, TM cells expressing myocilin at low levels and at high levels after Dex treatment for 5 days. TGF-β2 is the major isoform of the TGF-β family found in ocular tissue and mediates abnormal extracellular matrix (ECM) remodeling in TM cells, leading to increased IOP and glaucoma 13. Previous studies have detected that the MYOC Y437H mutant was misfolded in the ER, which resulted in ER stress and TM cell death 14. In order to test whether TGF-β2 has effects on MYOC Y437H mutant induced cellular abnormalities, TM cells were infected with adenovirus expressing either WT MYOC or Y437H mutant. These two TM cell groups were then exposed to TGF-β2 (50ng/ml) for 48 h, after which myocilin expression as well as the expression of two ER stress markers (GRP94 and CHOP) were determined. As shown in Fig. 4B and C, myocilin expression was increased in the MYOC Y437H mutant group when compared with TM cells expressing WT MYOC. Furthermore, only expressing of MYOC Y437H mutant, but not WT MYOC, resulted in the elevation of ER stress markers GRP94 and CHOP. Interestingly, exogenous TGF-β2 treatment signifycantly increased the expression of myocilin as well as GRP94 and CHOP in TM cells expressing MYOC Y437H mutant. Thus, these data indicated that TGF-β2 can enhance the expression of myocilin and ER stress in TM cells expressing MYOC Y437H mutant.

### TGF-β2 increases MYOC Y437H mutation induced cell death

**Figure 5:**
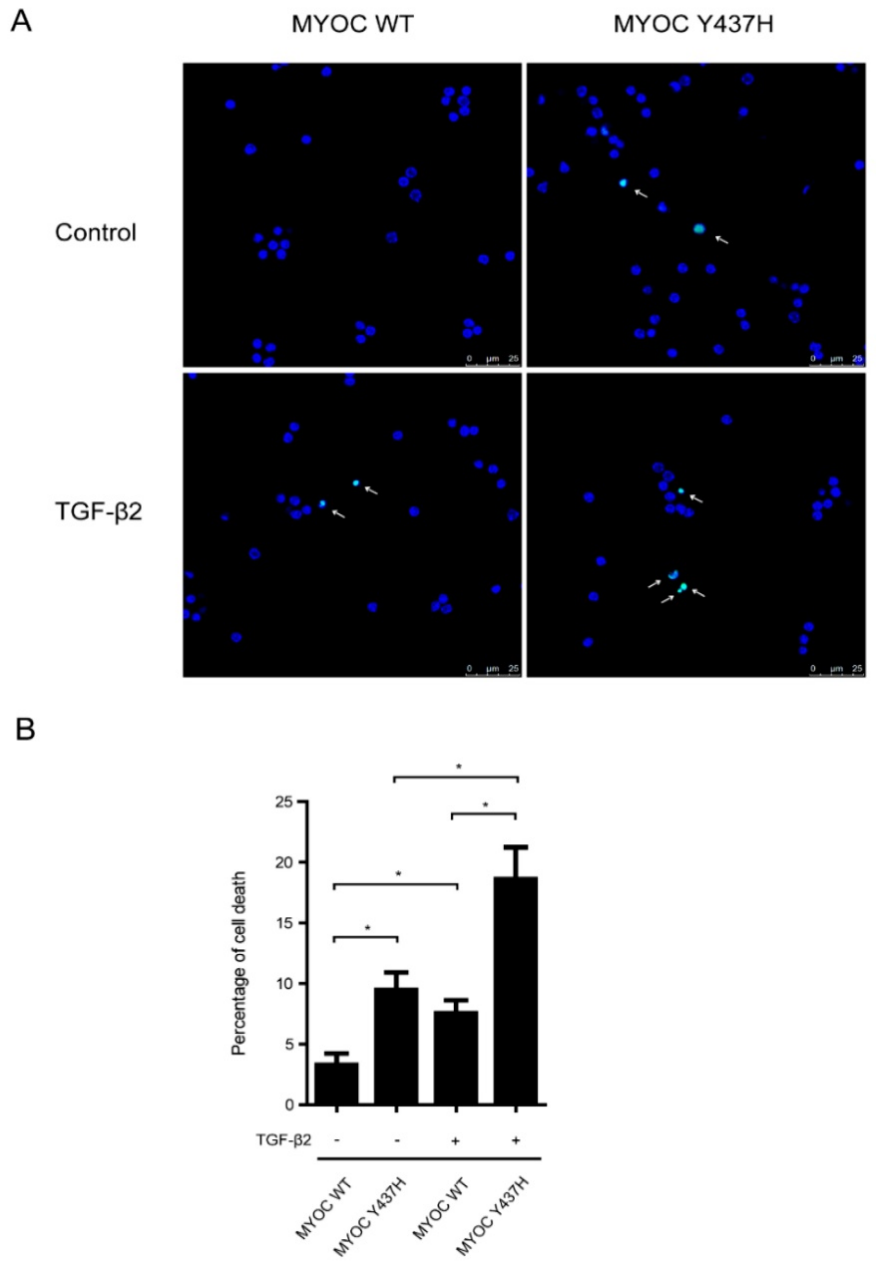
** TGF-β2 increases MYOC Y437H mutation induced cell death.** (A) TM cells were overexpressed with WT MYOC or Y437H mutant first, then exposed to TGF-β2 (50ng/ml) for 48h. Cell death was detected by TUNEL staining. (B) Quantification of the percentage of TUNEL positive cells.

We next sought to determine the interplay between TGF-β2 and MYOC Y437H mutation in induced cell death. To do so, human TM cells expressing either WT MYOC or Y437H mutant were treated with or without TGF-β2 (50 ng/ml) for 48 h, after which TUNEL staining was used to detect cell death (Fig. 5A and B). These results showed that when compared with WT MYOC-expressing TM cells, cells expressing the MYOC Y437H mutant saw a marked increase in cell death. More specifically, the percentage of TUNEL positive cells in these two groups was 3.3%±0.9 and 9.5%±1.5, respectively. Moreover, exogenous TGF-β2 significantly increased the percentage of cell death both in the WT MYOC and Y437H mutant groups (increased to 7.5%±1.1 and 18.6%±2.6, respectively). Taken together, these results demonstrated that TGF-β2 enhanced susceptibility to cell death in MYOC Y437H mutant cells.

## Discussion

Glaucoma is an ocular disease with high incidence, unknown etiology, and high genetic heterogeneity. Until now, studies have reported more than 70 *MYOC* mutation sites in glaucoma patients. The Myocilin protein is encoded by the *MYOC* gene and is expressed widely in the body, including in skeletal muscle, heart, lung, pancreas, corpus ciliare, trabecular meshwork, and retina 15. Current international work holds the *MYOC* mutation rate to be approximately 2-6% in POAG patients 5, 16. The *MYOC* Y437H mutation is located in the third exon of *MYOC* and was first reported in 1998 17. This study found that the probability of glaucoma in a family carried Tyr437His mutation was about 10^-12^ to 10^-14^, which was far higher than the risk presented in the normal population. Furthermore, the time of glaucoma onset in patients with this Tyr437His mutation was much earlier than in patients with the Gln368X mutation, along with a generally higher corresponding IOP. This phenotype may be related to the dominant negative effect of the *MYOC* mutation. Additional work showed that the MYOC Y437H mutant was misfolded and aggregated in the endoplasmic reticulum, inducing abnormal ECM accumulation in the ER and unfolded protein response (UPR) activation 18.

The normal function of the myocilin protein and pathogenic mutations of *MYOC* in glaucoma is not well understood. *In vitro* studies have shown that myocilin expression is related to IOP, glucocorticoid, and TGF-β2 19, 20, and its expression was related to the synthesis and flow of aqueous humor. Mouse work by Kim *et al.* found *MYOC* gene deletion did not change either the intraocular pressure or ocular morphology, which suggested that ability of the *MYO*C mutation to induce POAG may occur through a gain-of-function effect rather than deficient myocilin expression 4. MYOC glaucoma-related mutations may activate inflammatory responses by activating the IL-1/NF-κB pathway. This would, in turn, be inhibited by the expression of wild-type MYOC 21. In this study, we detected a c.1309T>C (p. Y437H) mutation in the *MYOC* gene in a Chinese POAG family. In this family, we found that although III:10 aged 13 carried the Tyr437His mutation and had high IOP, III:10 was not ultimately diagnosed with glaucoma. This might be because III:10 had not yet reached the age of onset. We further defined decreased myocilin levels and simultaneous increased TGF-β2 levels in the aqueous humor of *MYOC* Y437H mutation carriers. This finding provided a direct link between the *MYOC* Y437H mutation and TGF-β2 production in aqueous humor, which suggests that mutant MYOC (Y437H) protein may activate the inflammatory response in the pathogenesis of POAG by modulating TGF-β2.

As the major isoform of TGF-β family in ocular tissue, TGF-β2 is reported to play important roles in the process of glaucoma via the modulation of ECM synthesis, secretion, and degradation in the human TM cells 22-24. Previous observations have indicated that TGF-β2 concentration is significantly higher in patients with either open-angle glaucoma (OAG) or POAG 11, which mark TGF-β2 as a novel therapeutic target for POAG. In fact, targeting TGF-β isoforms and/or related receptors has proven promising therapeutic targets in a glaucoma animal model 25. For instance, in a rabbit model of glaucoma filtration surgery, subconjunctival injection of anti-TGF-β2 antibody CAT-152 significantly improved glaucoma surgery outcomes and reduced scarring 26. However a Phase III study of subconjunctival injection of CAT-152 failed to prevent the failure of primary trabeculectomy 27. Recently, the first human Phase I trial demonstrated that intravitreal injection of an antisense oligonucleotide ISTH0036 that selectively targets the TGF-β2 at the end of trabeculectomy is safe and potentially effective 28. Collectively, signs of potential therapeutic value have been observed in these studies. However, further research aimed at elucidating the relationship between glaucoma and inflammatory response mediated by TGF-β2 are needed, and the results from these studies may help optimize future therapeutic strategies targeting TGF-β2.

## Figures and Tables

**Table 1:** Primers sequences of MYOC gene

Primer	Primer sequence (5′-3′)
*MYOC*-1F	ctctgtcttcccccatgaag
*MYOC*-1R	agcaggtcactacgagccata
*MYOC*-2F	agtgtagtctcggctcacagc
*MYOC*-2R	tctgctcccagggaagttaat
*MYOC*-3-1F	tccgcatgatcattgtctgt
*MYOC*-3-1R	aatgggatggtcagggtctt
*MYOC*-3-2F	catccgtaagcagtcagtcg
*MYOC*-3-2R	tcccacaaagttcaaggaaga

**Table 2:** Clinical Data of Y437H mutation carriers in this family (n=7)

Pedigree number	Gender	Age at study (years)	Highest IOP NCT OD/OS (mm Hg)	IOP at study NCT OD/OS (mm Hg)	Age at diagnosis (years)	Operation eye/age (years)	BCVA OD/OS	C:D ratio OD/OS	Visual field damage OD/OS
I:1	Male	82	48/50	33/30	39	OU/40	LP/40 cm	1.0/1.0	NA
II:3	Male	53	45/48	17/14	32	OU/39	HM/40 cm	1.0/1.0	NA
II:5	Male	50	39/40	16/19	28	OS/34	0.2/0.8	0.9/0.6	Severe/Moderate
II:7	Female	48	50/53	25/20	22	OU/26	0.5/0.6	0.8/0.9	Moderate/Moderate
III:5	Male	30	42/40	12/10	23	OD/27	0.1/0.3	0.9/0.9	Severe/Moderate
III:9	Female	22	44/45	13/14	16	OS/20	0.04/0.1	0.8/0.9	Severe/ Severe
III:10	Female	13	22/23	17/18	―	―	1.0/1.0	0.2/0.2	Normal

IOP, intraocular pressure; NCT, non-contact tonometer; OD, oculus dexter; OS, oculus sinister; LP, light perception; HM, hand move; BCVA, best corrected visual acuity; C:D ratio, cup/disc ratio; NA, patient was not available for examination

## Abbreviations

ER: endoplasmic reticulum
POAG: Primary open-angle glaucoma
TM: trabecular meshwork
Dex: dexamethasone
ECM: extracellular matrix
IOP: intraocular pressure
NCT: non-contact tonometer
OD: oculus dexter
OS: oculus sinister
BCVA: best corrected visual acuity
C:D ratio: cup/disc ratio
